# Predicting Suitable Areas for African Swine Fever Outbreaks in Wild Boars in South Korea and Their Implications for Managing High-Risk Pig Farms

**DOI:** 10.3390/ani13132148

**Published:** 2023-06-29

**Authors:** Ju Hui Choi, Hun Namgung, Sang Jin Lim, Eui Kyeong Kim, Yeonsu Oh, Yung Chul Park

**Affiliations:** 1 College of Forest & Environmental Sciences, Kangwon National University, Chuncheon 24341, Republic of Korea; jh_003@naver.com (J.H.C.);; 2 Ecological Survey Division, Korea National Park Research Institute, Wonju 26441, Republic of Korea; 3 College of Veterinary Medicine & Institute of Veterinary Science, Kangwon National University, Chuncheon 24341, Republic of Korea

**Keywords:** wild boar, carcass, African swine fever, ASF, MaxEnt, Shortest path betweenness centrality

## Abstract

**Simple Summary:**

Predicting suitable areas and paths for African swine fever (ASF) outbreaks is crucial for early detection and removal of ASF virus (ASFV)-infected carcasses in ASF-prevalent regions, as well as for the establishment of preemptive quarantine measures in ASF-free regions. In this study, we utilized the MaxEnt model and shortest-path betweenness centrality to predict areas with a high likelihood of ASF outbreaks in wild boars while also identifying individual pig farms and pig farm sectors at high risk of ASFV spillover from wild boars. The results of this study are intended to help to save time and cost in searching for carcasses by specifying the search range for ASFV-infected wild boar carcasses. Additionally, the study’s findings could help pig farms at high risk of ASFV spillover establish preemptive quarantine measures such as reinforcing biosecurity inside the farms and routinely searching for carcasses around the farms.

**Abstract:**

African swine fever (ASF) is a highly contagious disease affecting domestic pigs and wild boars, with no effective vaccine or treatment available. In South Korea, extensive measures have been implemented to prevent ASF transmission between wild boars and ASF spillover from wild boars to pig farm sectors, including the search for ASF-infected carcasses in mountainous forests and the installation of fences across wide areas of these forests. To determine the priority search range for infected carcasses and establish pig farm-centered quarantine measures, it is necessary to predict the specific path of ASF outbreaks in wild boars and identify pig farms at high risk of ASF spillover from wild boars. Here, we aimed to predict suitable areas and geographical paths for ASF outbreaks in wild boars using the MaxEnt model and shortest-path betweenness centrality analysis. The analysis identified a high frequency of ASF outbreaks in areas with a suitability value ≥0.4 on the suitability map and in areas within a 1.8 km range from the path on the shortest-path map, indicating these areas were high-risk zones for ASF outbreaks. Among the 5063 pig farms analyzed, 37 were in the high-risk zone on the suitability map, 499 were in the high-risk zone on the shortest-path map, and 9 were in both risk zones. Of the 51 pig farm sectors with a dense distribution of pig farms (kernel density ≥ 8), 25 sectors were in contact with or partially overlapped the high risk zone on the suitability map, 18 sectors were located within the high risk zone on the shortest-path map, and 14 sectors were located within both risk zones. These findings aided in determining the priority range for searches for wild boar carcasses and enabled the establishment of preemptive ASF prevention measures around the pig farming sectors that are at risk of ASF spillover from wild boars.

## 1. Introduction

African swine fever (ASF) is a highly contagious disease affecting domestic pigs and wild boars, caused by infection with the African swine fever virus (ASFV) [1]. Currently, no effective vaccine or treatment has been developed for the disease, and lethality can reach 100% [2,3,4].

ASFV can survive within wild boar populations with a low incidence rate of less than 5% [5] and can maintain a chronic infection with low-virulence ASFV isolates [6]. Therefore, wild boars can act as a reservoir for ASFV [7] and pose a constant risk of ASFV spillover in domestic pigs [8,9,10]. ASF outbreaks in wild boars have been highly related to those on domestic pig farms in the EU and Russia [7,11,12,13]. ASFV spillover from wild to domestic pigs has frequently been reported in small backyard pig farms with inadequate fences and relatively low biosecurity [9,14,15,16,17]. In South Korea, ASFV is unlikely to be transmitted through direct contact between wild boars and domestic pigs because domestic pigs are raised in pigsties enclosed in buildings surrounded by fences. Thus, ASFV could be transmitted to domestic pigs via humans [18,19,20] or wildlife [5,21,22,23] that have come into contact with ASFV-infected wild boar carcasses and their remnants around pig farms. Thus, predicting the possibility and location of wild boar ASF outbreaks near pig farms is essential to establishing the specific range of preemptive quarantine measures for pig farms.

ASFV is a stable DNA virus that can survive for at least 11 days in feces, approximately 15 weeks in corrupted serum, and months in the spinal cord [24,25]. Although it is not transmitted by direct contact, farmers who come into contact with contaminated environmental substances, such as soil and water contaminated during the decay process of ASFV-infected wild boar carcasses, may introduce ASFV into pig farms [5,11,26]. Wild animals that come into contact with carcasses are also suspected carriers of ASFV [5,21]. Although there is no direct evidence that wild animals can transmit ASFV, there is considerable evidence that wild animals such as wild boars, rats, raccoons, leopard cats, and birds come in contact with or consume wild boar carcasses [21,22,23]. Thus, rapid detection and removal of ASFV-positive wild boar carcasses is essential to preventing the spread of ASF among wild boars and the spillover of ASF into the pig farm sector [27].

Identifying the areas and paths suitable for the transmission of ASF in wild boars plays an important role in selecting fence installation areas, identifying the range of ASFV infection detection, setting up hunting areas to control the population of wild boar, and establishing intensive quarantine measures for pig farms at high risk of ASF.

Ecological niche modeling (ENM) is the process of exploring non-random relationships between known species occurrence locations and corresponding environmental variables [28,29]. ENM can be applied to explain and predict present and future spatiotemporal distribution of species [30,31]. It has been widely used in the epidemiology of infectious diseases and wildlife habitat management [31,32] to predict the geographic distribution of pathogens [33,34,35], parasites [36], hosts [37,38,39,40], and vectors [41,42,43,44] and to explore biotic and abiotic conditions associated with the locations of disease outbreaks [29,31,33,38,41,43,45]. Maximum entropy (MaxEnt) is a general purpose ecological niche model that predicts a species’ geographic distribution based on occurrence data alone [46,47]. The MaxEnt model uses environmental data to predict the distribution of targeted species across geographic space and time [46]. It has also been used to predict disease outbreak risks [29,48,49,50].

Connectivity analyses, such as shortest-path betweenness centrality (BC), current flow models, and network flow, have been widely used not only to conserve regional habitat connectivity [51,52,53] but also to predict the spatial spread route of pathogens or hosts [54]. Shortest path BC analysis identifies one or several shortest paths (a minimal network of linkages) that connect each pair of nodes on a graph and counts the number of shortest paths in which a node is included [52,55]. It has been applied in various fields, ranging from connecting wildlife habitats [52,56,57,58] to predicting the path of wildlife disease or vector spread [59,60].

**Figure 1:**
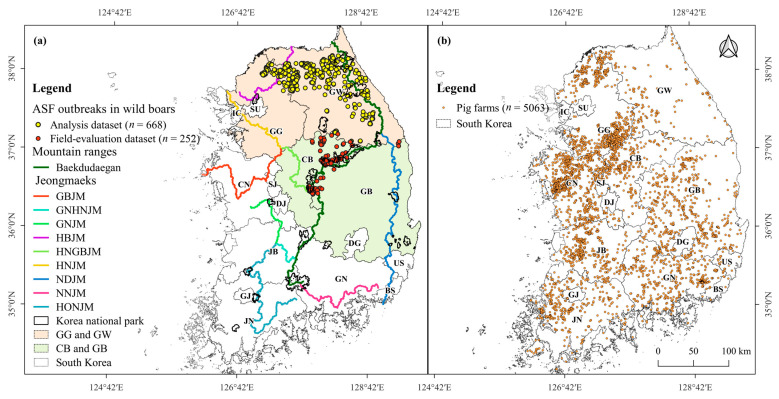
Geographical distribution map of (**a**) 920 wild boar ASF outbreaks (colored circles) and (**b**) 5063 pig farms. Among the 920 ASF outbreaks, GPS locations of 668 cases (yellow-circled dots) in GW and GG (light orange-shaded local regions) were used in the analysis dataset, and GPS locations of 252 cases (red-circled dots) in CB and GB (light green-shaded local regions) were used for the field-evaluation dataset. The mountain ranges in this region include Baekdudaegan, which stretches across the entire Korean Peninsula, also known as the geographical backbone of the Korean peninsula, and nine Jeongmaeks, which are mountain ranges that branch off from the Baekdudaegan (Figure S1). The Korean administrative district boundary shapefile was provided by GIS Developer [61].

In South Korea, on September 16, 2019, the first ASF outbreak was reported on a pig farm in Gyeonggi-do (GG), South Korea. Shortly thereafter, on October 2, 2019, ASFV was first detected in a wild boar carcass near the Demilitarized Zone (DMZ) in the northern part of GG. Since then, the virus has spread widely throughout the eastern part of GG and most parts of Gangwon-do (GW), which is located east of GG (Figure 1a). On 19 November 2021, ASF was reported in wild boars in Chungchungbuk-do (CB) and subsequently in Gyeongsangbuk-do (GB), the local region bordering the southern part of GW (Figure 1a). To prevent ASF spillover from wild boars into pig farm sectors, a 2809 km-long fence has been installed within mountainous forests and at the forest boundary, and an active search for wild boar carcasses has been conducted in a wide range of mountainous forests by the Ministry of the Environment, local governments, and the National Park Service.

Previous studies predicting the spread of ASF in wild boars have primarily focused on scenarios involving the range of expansion of ASF within wild boar populations [1,62,63,64] and the correlation of ASF outbreaks between wild boars and domestic pigs [31,48,50,65,66]. However, few studies have predicted specific paths of ASF outbreaks in ASF-free areas where ASF is expected to spread in the near future. Furthermore, in areas where ASF has not yet spread, the potential risk of ASF spillover from wild boars to pig farm sectors has not been predicted by linking the expected paths of wild boar ASF outbreak to the geographical distribution of pig farms. This study predicts specific pathways of ASF spread through wild boars in ASF-free areas as well as ASF-contaminated areas using modeling approaches and field evaluation of ASF outbreak in wild boars.

The study makes an important contribution to the field by facilitating preemptive ASF quarantine measures in two key aspects. First, it aids in determining the priority range of searches for wild boar carcasses. Second, it enables the establishment of preemptive ASF prevention measures around pig farm sectors that are at potential risk of ASF spillover from wild boars in ASF-free areas.

To this end, we predicted potentially suitable areas for ASF outbreaks in wild boars by combining environmental data with the locations of ASF outbreaks, using the combined MaxEnt model and GIS-based methodology. We then predicted the geographical path of ASF outbreaks through shortest-path BC analysis. We also identified pig farm sectors with high densities of pig farms using the kernel density estimate (KDE). Finally, we selected individual pig farms and pig farm sectors in the high-risk zone of ASF outbreaks by linking the geographical distribution of pig farms to potential spread areas of ASF in wild boars predicted by the models in ASF-contaminated- and ASF free-areas.

## 2. Materials and Methods

### 2.1. Status of ASF Outbreaks and Dataset Construction

Two datasets were constructed for the analysis and evaluation: an analysis dataset and a field-evaluation test dataset containing the GPS locations of ASF outbreaks in wild boars (Figure 1a). The analysis dataset consisted of GPS locations of ASF outbreaks in the local regions of GG and GW, where ASF was widespread in the wild boar population. The analysis dataset was used for the MaxEnt model and shortest-path betweenness centrality (BC) analysis to predict suitable areas and shortest paths for ASF outbreaks in wild boars. The field-evaluation dataset comprised GPS locations of ASF outbreaks in the local regions of CB and GB, where ASF spread had newly commenced; ASF outbreaks have been restricted in some areas of these local regions. The field-evaluation dataset was used to evaluate suitable areas and paths of ASF outbreaks predicted using the MaxEnt and shortest-path BC analyses.

To construct the analysis dataset, the GPS locations of 1567 ASF outbreaks reported in wild boars in GW and GG until 19 November 2021 were selected. Among them, 899 cases were found in the Civilian Control Area (CCA) south of the DMZ between South and North Korea. However, environmental information related to these 899 cases, such as land cover map and forest type map, was unavailable (Figure S1). Therefore, the GPS locations of 668 cases were included in the analysis. The field-evaluation dataset contained the GPS locations of 252 ASF outbreaks reported in wild boars from 20 November 2021 to 14 November 2022, in CB and GB (Figure 1a). The GPS locations of ASF outbreaks in wild boars were provided by the Ministry of Agriculture, Food, and Rural Affairs website [67].

We constructed a pig farm dataset for KDE to identify the geographical distribution and density of pig farms. The dataset included the GPS locations of 5063 pig farms as of 3 February 2022 (Figure 1b) and was provided by the Korea Animal Health Integrated System (Figure 1b).

### 2.2. Environmental Variables

Eleven environmental variables (Table 1 and Figure S2) were used for the analysis. These variables were categorized into forest (*n* = 4), topographic (*n* = 4), and anthropogenic (*n* = 3).

(1) Forest type (frtp), age class (agcl), diameter at breast height of trees (dbht), and crown density (crde) were extracted from a 1:5000 forest map (shapefile) obtained from the Korea Forest Service. The extracted data were converted into a raster file with a 90-m resolution using the Polygon-to-Raster Tool in ArcMap 10.3.1.

(2) The topographic variables of elevation (elev), slope (slop), aspect (aspe), and water, which affect habitat selection in terrestrial animals [68], were extracted from a 90-m resolution digital elevation model (DEM) provided by the National Geographic Information Institute of the Republic of Korea. The slope and aspect variables were calculated using the Slope and Aspect Tools in QGIS Desktop 3.22.11. For the water variable (water), we used the Euclidean distance tool in ArcMap 10.3.1 to generate the distance to water (raster) with a resolution of 90 m based on a shapefile provided by the Water Resources Management Information System (WAMIS) of Republic of Korea, which contains data on all water systems in the country, from streams to lakes.

(3) The anthropogenic environmental variables included the distance from the settlement (setl), plow (plow), and road (road). Settlement and plow (shape files) were extracted from a land cover map (1:5000) provided by the Ministry of the Environment of the Republic of Korea. The data for road (shapefile) was extracted from the Korean Road Network Data provided by the Ministry of Land, Infrastructure, and Transport. The distances from the three variables (raster) were created with 90 m resolution using the Euclidean Distance Tool in ArcMap 10.3.1. All variables with a 90 m resolution were converted to Ascii files for use as input data for MaxEnt.

Correlation and collinearity were analyzed to produce a reliable and unbiased model of species distribution. We evaluated the correlations between the 11 environmental variables using Spearman’s correlation coefficient in R version 4.2.2. None of the variables showed a correlation (absolute value less than 0.75) or multicollinearity. Therefore, all 11 variables were included in the analysis. The relative importance of the variables was evaluated using the Jackknife test in MaxEnt [69].

### 2.3. Data Analysis

#### 2.3.1. Predicting Suitable Areas for ASF Outbreaks

The MaxEnt model was used to predict suitable areas of ASF outbreaks using MaxEnt 3.4.4 software [46]. We used the overview, data, model, assessment, and prediction method (ODMAP protocol) described in [70] to develop suitability models for ASF outbreaks in South Korea (Table S1). Duplicate records (multiple records in the same grid cell) were removed by default command “Remove duplicate presence records,” resulting in 591 locations for the MaxEnt model out of the 668 ASF outbreak locations in the analysis dataset. Data sets for testing and training were not spatially autocorrelated (Moran’s I test, *p*-value: 0.973) and therefore all occurrence records were retained for spatial analysis [71]. To correct for sampling bias, 20,000 background points were randomly selected and used, of which 80% (473) were used for training and the remaining 20% (118) were used for testing. All other parameters were set to their default values (maximum iterations = 500, convergence threshold = 0.00001, default prevalence = 0.5) [72]. The final prediction model for the ASF outbreak was selected as the mean model from ten bootstrap replicates. Model performance was evaluated based on the AUC (Area Under the ROC Curve) value calculated from the area under the Receiver Operating Characteristic Curve [73] and TSS (True Skill Statistic) value [74]. With respect to MaxEnt model performance, the AUC value ranges from 0–1, where an AUC < 0.7 denotes poor model performance, 0.7–0.9 denotes moderately useful model performance, and >0.9 denotes excellent model performance [73,75]. The TSS values ranges from −1 to 1, where −1 to 0.4 = poor, 0.4 to 0.5 = fair, 0.5 to 0.7 = good, 0.7 to 0.85 = very good, 0.85 to 0.9 = excellent, 0.9 to 1 = almost perfect to perfect [74,76,77]. To produce suitability maps for the ASF outbreak, a logistic link function was used to yield a suitability index between 0 and 1 in MaxEnt. The continuous habitat suitability index was further divided into five classes (0–0.2: very low suitability, 0.2–0.4: low suitability, 0.4–0.6: medium suitability, 0.6–0.8: high suitability, 0.8–1: very high suitability) [78,79].

#### 2.3.2. Predicting the Shortest Path for an ASF Outbreaks

Shortest-path BC analysis was performed using the Connectivity Analysis Toolkit (CAT) 1.3.2 [52]. First, HexSim 4.0.18 was used to create a workspace (grid file) covering South Korea, which consisted of 32,629 hexagons with an area of 5 km^2^ each [52]. Subsequently, the suitability map produced by MaxEnt was loaded into the workspace. Each hexagon contained 617 pixels, with an area of 0.0081 km^2^ each. The suitability score of each hexagon was calculated by summing the suitability index values of all pixels within the hexagon extracted from the suitability map. Hexmap, a lattice of 32,629 hexagons with suitability scores, was loaded into CAT 1.3.2 to generate the graph. After setting Edge List-Distance (Betweenness) as the graph type, a graph file was produced and used to calculate the shortest-path BC with a probability of 0.05 as the default value. The resulting shortest-path BC routes were classified into three categories (low, medium, and high) using Jenks Natural Break in QGIS Desktop 3.22 [80]. The density of the crossing points and routes of the shortest path, BC, was estimated using the Kernel Density Estimation Tool in QGIS Desktop 3.22 [81]. KDE is a spatial analysis technique that accounts for the relative locations of features to each other [81].

#### 2.3.3. Pig Farm Density

To identify areas with high concentrations of pig farms throughout South Korea, the kernel density of 5063 pig farm locations was estimated using the Kernel Density Estimation Tool in QGIS Desktop 3.22. According to the standard operating procedure (SOP) for ASF [82], three types of ASF management zones have been established: a control zone within a 500 m radius from an infected farm, a protection zone within a 3 km radius, and a surveillance zone within a 3–10-km radius. In the KDE analysis, the protection zone was selected as the buffer among the three criteria for ASF management presented in the SOP. Therefore, a buffer with a radius of 3 km was set around the location of each farm, and the resolution of the pixels on the output map was set to 100 m.

## 3. Results

### 3.1. Suitable Areas for ASF Outbreaks

**Figure 2:**
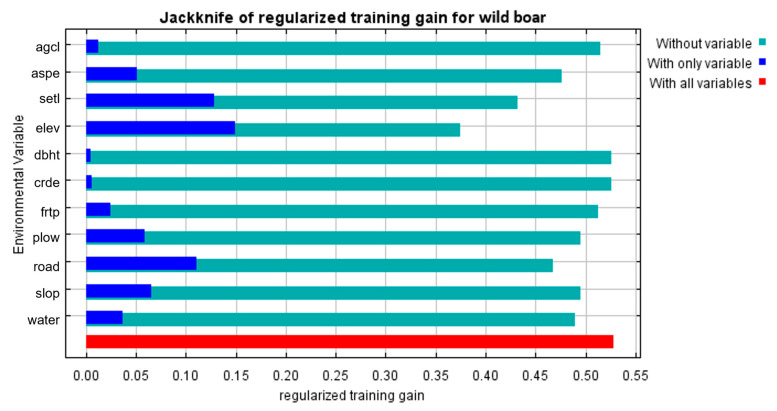
Jackknife test for regularized training gain of individual environmental variable importance (blue bars) relative to all environmental variables (red bar) for the MaxEnt model.

**Figure 3:**
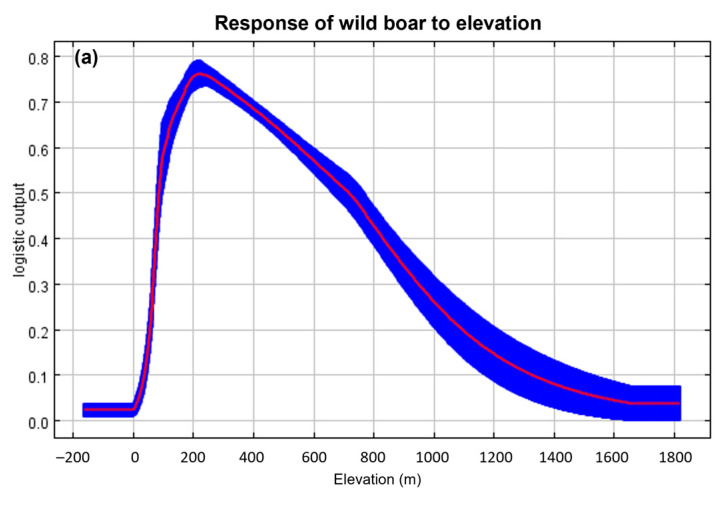

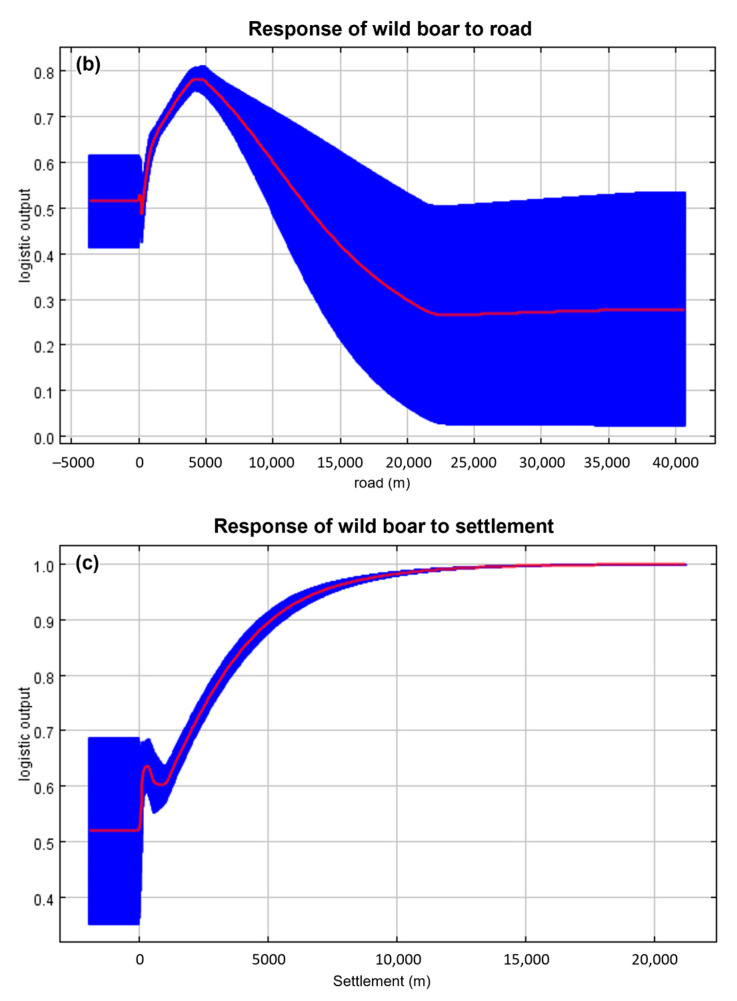
Response curves of the three highest contributing variables (**a**–**c**) for predicting suitable areas for ASF outbreaks. The curves show the mean response of the 10 replicate MaxEnt runs (red) and the mean ± standard deviation (blue, two shades for categorical variables).

The average test AUC for the 10 replicate runs in the MaxEnt model was 0.793 (*n* = 10, standard deviation = ± 0.008, range = 0.784–0.809) (Figure S3) and that of TSS value was 0.4 (standard deviation = ± 0.04, range = 0.37–0.45). The regularized training gains of the Jackknife tests indicated that the variables of elevation, settlement, and road made the highest contribution to the model; the four variables of aspect, slope, forest type and plow contributed moderately, and age class; and diameter at breast height of trees and crown density (crde) contributed weakly (Figure 2). According to the percentage contribution, elevation was the most important variable, contributing 33% to the model prediction, followed by distance from road (16%) and distance from settlement (15.6%), which accounted for 64.6% of the total contribution (Table S2). Permutation importance also showed that the three variables were important, accounting for 56.7% of the total importance (Table S2). In the response curves of the top three contributing variables, the probability of an ASF outbreak increased between 100 m and 550 m elevation (Figure 3a) and between 700 m and 10,000 m distance from the road (Figure 3b). The probability of an outbreak increased at a distance of 150 m from the settlement, decreased slightly from 500 m to 1000 m, and increased again as the distance increased (Figure 3c).

**Figure 4:**
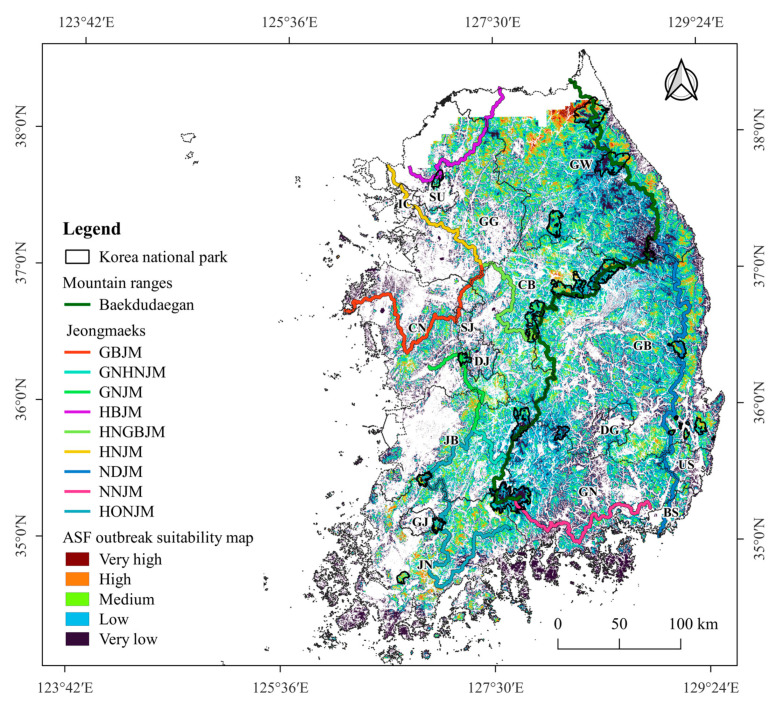
Suitable areas for ASF outbreaks predicted using the MaxEnt model.

A forest area of 52,790 km^2^, excluding forests in the CCA and islands, was included in the MaxEnt model and the shortest-path BC analysis. Of the 52,790 km^2^ of forests included in the analysis, the MaxEnt model predicted that 17,193 km^2^ had a very low suitability for ASF outbreaks, 21,071 km^2^ had low suitability, 11,647 km^2^ had medium suitability, 2682 km^2^ had high suitability, and 197 km^2^ very high suitability. The total area with a suitability value ≥ 0.4 was 14,526 km^2^, accounting for 28% of the analyzed forests. The suitable area with a suitability value ≥ 0.4 was mainly distributed in the northern region in GW and near Baekdudaegan (Figure 4). Additionally, a high probability of an ASF outbreak was predicted near an eastern mountain range (NDJM) in the eastern part of GB, which extends southward from Baekdudaegan.

In CB and GB, a forest area of 16,469.1 km^2^ was included in the MaxEnt analysis. The total area with a suitability value ≥ 0.4 was 4824.2 km^2^, which accounted for 29.3% of the analyzed forest area in CB and GB. Of the 252 ASF outbreaks in the field-evaluation dataset, 173 cases (68.6%) were located in areas with a suitability value ≥ 0.4 (Table 2). The number of ASF outbreaks per km^2^ in areas with a suitability value ≥ 0.4 was 0.036. However, the number of ASF outbreaks in areas with suitability values < 0.4 was only 0.007, indicating a 5.1-fold increase in the number of outbreaks in areas with higher suitability area than in those with lower suitability.

### 3.2. Shortest-Path of ASF Outbreak

**Figure 5:**
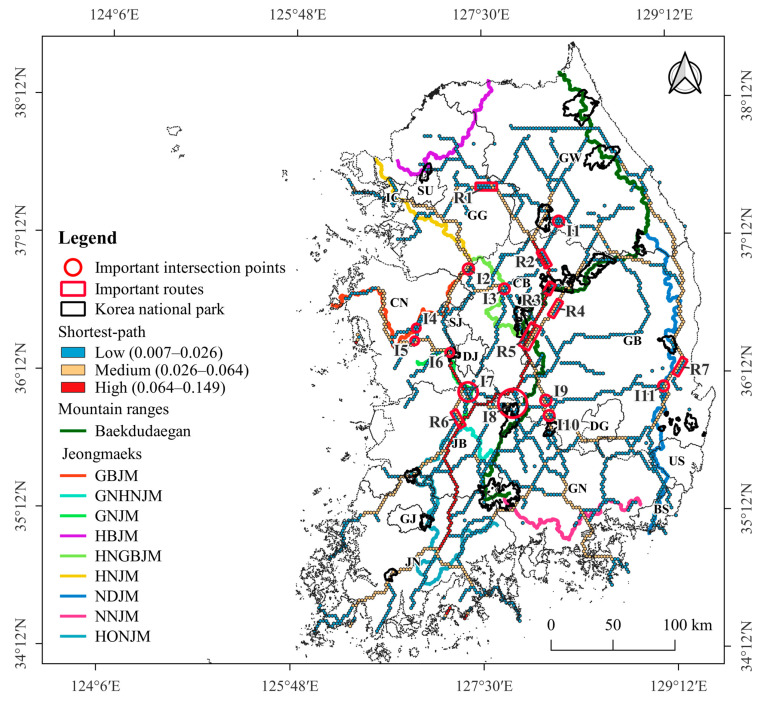
Shortest paths for ASF outbreaks. The 7 locations (R1–R7) on the shortest paths and 11 locations (I1–I11) at the intersections of the shortest paths indicates locations with a kernel density value of 1.67 or higher.

The shortest-path BC values ranged from 0.00–0.149 and were classified into three categories, strong (0.064–0.149), medium (0.026–0.064), and weak shortest-path (0.007–0.026), using the Jenks natural breaks method. The shortest paths were primarily generated along the mountain ranges. A strong shortest path (marked in red) was produced from the north to the southwest in the middle of South Korea. In the eastern part, a medium-shortest path (marked in yellow) was produced from the north to the south along Baekdudaegan and NDJM, which branches off from south of Baekdudaegan, east of GW and GB (Figure 5).

The KDE analysis revealed that the kernel density values of the center points of the hexagons (*n* = 2375) in the shortest paths ranged between 1–1.86. These values were classified into four levels (Low: 1.46–1.5, Medium: 1.5–1.56, High: 1.56–1.67, Very High: 1.67–1.86) using the Jenks natural breaks method. The analysis predicted a very high kernel density value of 1.67 or higher (VHKD) for 7 parts (R1–R7) and 11 intersections (I1–I11) on the shortest paths (Figure 5); 7 of the 18 VHKD locations were close to national parks: Chiaksan National Park (I1), Woraksan National Park (R3), Songrisan National Park (R5), Deogyusan National Park (I8), Gayasan National Park (I10), Gyeryongsan National Park (I6), and Juwangsan National Park (R7).

All of 252 ASF outbreaks in the field evaluation dataset were distributed at distances of 0.25–9.2 km from the shortest paths, with an average range of 1.8 (standard deviation = ± 1.3 km). Among them, 233 cases (92%) were found within a range of 3.6 km from the shortest-paths, with 167 cases (66%) within a 1.8 km range and 66 cases (26%) within a 1.8–3.6 km range from the paths. The number of ASF outbreak per km^2^ was 2.5 times higher within a 1.8 km range (0.032 cases/km^2^) from the paths compared to those within a 1.8–3.6 km range (0.013 cases/km^2^).

In the range of 1.8 km from the shortest paths, the number of ASF outbreaks per km^2^ was the highest in the strong shortest paths, followed by the medium and weak shortest paths (0.073 > 0.030 > 0.026). In contrast, within 1.8–3.6 km from the shortest paths, the number of ASF outbreak per km^2^ was higher in the medium, strong, and weak paths order (0.026 > 0.015 > 0.008) (Table 3).

### 3.3. Density of Pig Farms

**Figure 6:**
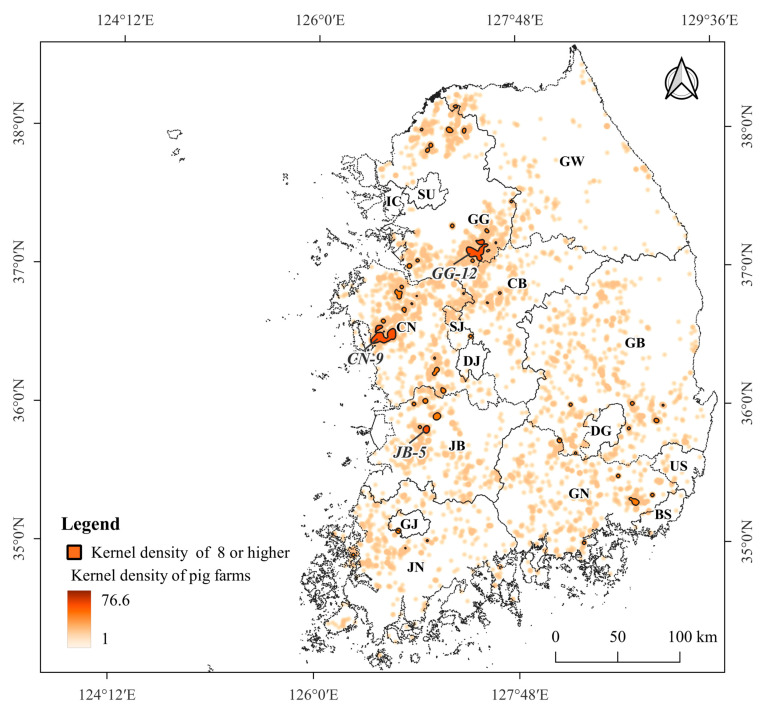
Kernel density values of the 5063 pig farms and 51 pig farm sectors with a density higher than the average kernel density (≥8): 15 pig farm sectors in GG, 1 in GW, 2 in CB, 12 in CN, 1 in SJ, 5 in JB, 3 in JN, 1 in GJ, 6 in GB, and 5 in GN. GG-12, CN-9, and JB-5 are large-scale sectors with over 100,000 heads each.

The kernel density values of the 5063 pig farms ranged from 1–76.6, with an average of 8 (standard deviation = ± 11.7). The kernel density values of 51 pig farm sectors, including 1319 pig farms, were higher than the average kernel density value (≥8). The number of pig farms with a kernel density of 8 or higher was the highest in GG (15 pig farm sectors). In GW, where ASF was the most widespread in wild boars, only one sector (GW-1) was identified in Cheorwon-gun, adjacent to GG (Figure 6). Three large-scale pig farm sectors (GG-12, CN-9, JB-5) with more than 100,000 heads each were distributed in GG, CN, and JB, respectively (Figure 6). The average shortest distance between individual pig farms was 457 m (standard deviation = ±315, range = 51–1677 m) in GG-12 with 216 pig farms, 444 m (standard deviation = ±284, range = 41–1758 m) in CN-9 with 285 pig farms, and 241 m (standard deviation = ±266, range = 49–1222 m) in JB-5 with 66 pig farms.

### 3.4. Pig Farms at High-Risk of ASF Outbreak

**Figure 7:**
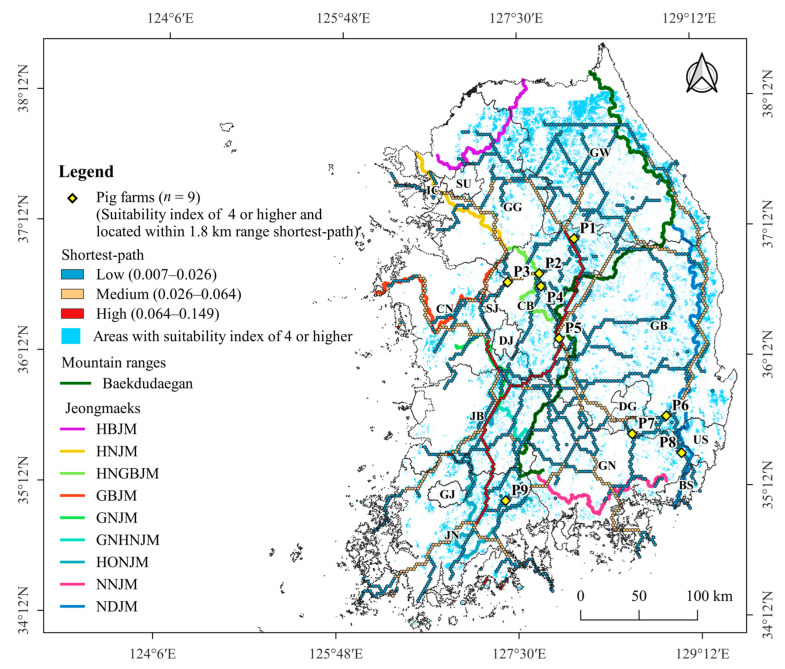
Geographic distribution of nine pig farms (P1–P9) located in both areas with a suitability value ≥ 0.4 and those within a range of 1.8 km from the shortest-path.

Of the 5063 pig farms, 37 were located in areas with a suitability value of ≥0.4 (Figure S4), 499 within a range of 1.8 km from the shortest-path (Figure S5), and 9 (P1–P9) in both areas with a suitability value ≥0.4 and those within a range of 1.8 km from the shortest-paths (Figure 7). The pig farms P2 and P4 were located 2.6 km and 4.9 km from HNGBJM, respectively, whereas P8 was located at a distance of 5.5 km from NDJM and P9 was 3.8 km from HONJM. The other five pig farms were located at distances greater than 10 km from the mountain ranges.

Of the 51 pig farm sectors with a kernel density greater than or equal to the average of 8, 18 were located within 1.8 km of the shortest path and 25 sectors were in contact with or partially overlapped areas with a suitability value ≥ 0.4 (Table 4). Among these sectors, 14 were both located within 1.8 km of the shortest path and were in contact with or partially overlapped with areas with a suitability value ≥ 0.4 (Table 4). Of the three large-scale sectors (GG-12, CN-9, and JB-5), CN-9 was adjacent to the shortest path and GG-12 was situated 0.6 km away from the path. Additionally, both of these sectors partially overlapped areas with a suitability value ≥ 0.4. (Table 4).

**Figure 8:**
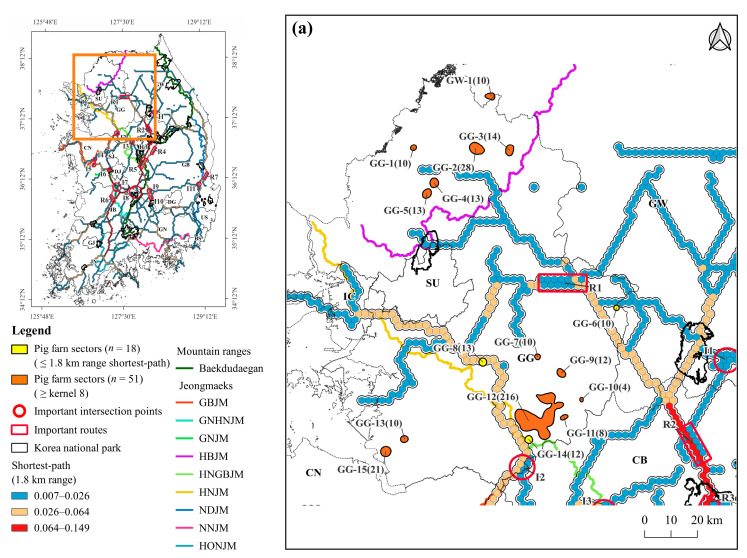

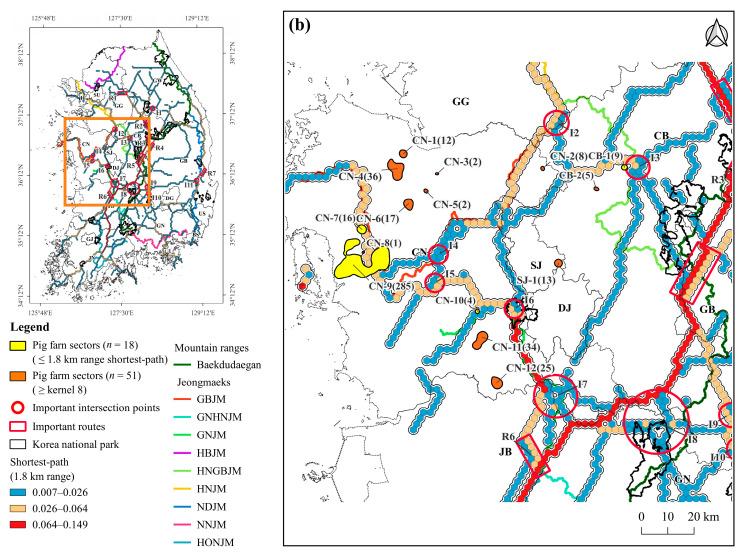

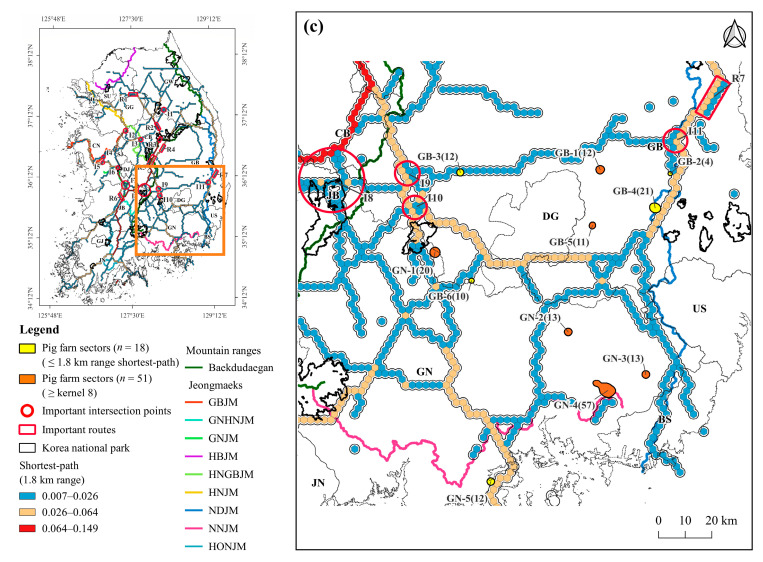

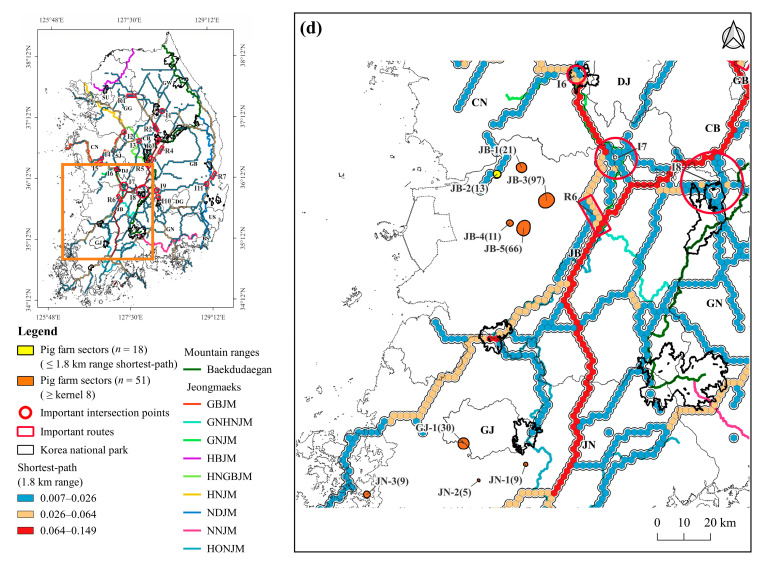
Geographic distribution of 51 pig farm sectors with a kernel density value equal to the average kernel density or higher (≥8). 18 pig farm sectors were located within 1.8 km of the shortest-path: (**a**) 5 sectors (GG-4, GG-6, GG-8, GG-12, GG-14) in GG; (**b**) 1 sector (CB-1) in CB and 4 sectors (CN-7, CN-8, CN-9, CN-10) in CN; (**c**) 4 sectors (GB-2, GB-3, GB-4, GB-6) in GB, and 3 sectors (GN-1, GN-4, GN-5) in GN; and (**d**) 1 sector (JB-2) in JB; 14 sectors also partially overlapped with or were adjacent to areas with suitability values of 0.4 or higher: (**a**) 5 sectors (GG-4, GG-6, GG-8, GG-12, GG-14) in GG; (**b**) 2 sectors (CN-7, CN-9) in CN; (**c**) and 4 sectors (GB-2, GB-3, GB-4, GB-6) in GB and 3 sectors (GN-1, GN-4, GN-5) in GN (also refer to Table 4 for partially overlapping areas or areas adjacent to those with a suitability value of 0.4 or higher).

Of the 18 pig farm sectors located within a 1.8-km range from the shortest path, 11 sectors overlapped or were adjacent to mountain ranges (Figure 8). In the GG and GW (Figure 8a) regions, GG-8 was adjacent to HNJM and GG-14 overlapped with HNGBJM. The large-scale pig farm sector GG-12 was situated only 0.6 km from the shortest path, and the eastern and southern sides of the sector are near HNJM and HNGBJM, respectively. In CB and CN (Figure 8b), CB-1 was adjacent to HNGBJM, CN-7 and CN-8 to GBJM, and CN-10 to GNJM. In the large-scale pig farm sector CN-9, the eastern side of the sector overlaps with the GBMJ. In GB and GN (Figure 8c), GB-2 and GB-4 overlapped with the NDJM. GN-4, a cluster of 57 pig farms with 97,584 heads, is adjacent to the NNJM. In JB and JN (Figure 8d), only one sector (JB-2) was located on the shortest path, but it was located at a considerable distance from the mountain range.

## 4. Discussion

### 4.1. Suitable Areas for ASF Outbreaks in Wild Boars

A notable feature of our study is that the model evaluation was not limited to self-evaluation metrics (AUC, TSS) alone (Figure S3), but also involved a comparison between the model results and the actual locations of wild boar ASF outbreaks observed in the field. Traditionally, evaluations of model performance have primarily relied on metrics such as AUC, TSS, and AIC [31,66,83,84,85]. In this study, a nationwide prediction model of ASF outbreaks in wild boars was established using the GPS coordinates of wild boar ASF outbreaks from widely affected areas, specifically GW and GG. To assess the effectiveness of the model, the model results were compared with the locations of wild boar ASF outbreaks (field-evaluation dataset) in GB and CB, where ASF in wild boars recently started spreading.

The MaxEnt model showed moderately useful performance in predicting suitable areas for ASF outbreaks in wild boars, with an average AUC value of 0.793 and TSS value of 0.4 for model reliability [75]. This was lower than the AUC value of the MaxEnt model built using the locations of ASF outbreaks in wild boars in North Sumatra Province, Indonesia (0.860) [86]. However, the field evaluation of our model provided meaningful results to predict potential risk area of ASF outbreak in wild boars. When the model results were evaluated with the field-evaluation dataset for CB and GB, 68.6% of 252 ASF cases were distributed in only 29.3% of areas with suitability value ≥ 0.4 in CB and GB, and the frequency of ASF outbreaks per km^2^ was higher in areas with the suitability values ≥ 0.4. These results indicate that the MaxEnt model using ASF outbreak locations (analysis dataset) in GW and GG has high predictive power with a suitability value of 0.4 or higher. The suitability maps generated in this study could be used to predict ASF outbreaks, particularly in areas in the early stages of the spread of ASF in wild boars.

A study in GG in South Korea used the MaxEnt model to predict the geographical distribution of ASF spread using the ASF outbreak locations [23]. In this study, settlement, elevation, and road were selected as important variables contributing to the model using the Jackknife test, similar to a previous study [23]. The AUC value obtained in this study was similar to that reported in a previous study (0.774). Both studies predicted highly suitable areas for ASF outbreaks in wild boars near the DMZ in the north of GG, Baekdudaegan in the east of GG, and the western part of GG. However, these results differed from the distribution of suitable areas for wild boar habitats based on wild boar occurrence locations (Baekdudaegan and south-eastern GG) [87] and hunting locations (south-central GG) [22]. This suggests that the spread area of ASF may differ from the preferred habitats of wild boar.

Integration with GIS and remote sensing (RS) has great potential to predict the spread of wildlife-borne diseases [13,88,89,90,91]. In order to further improve the results of the present study, additional environmental information obtained from RS is required for GIS analysis [91,92]. Meteorological data such as precipitation are known to be related to wild boar ASF outbreaks [93]. Therefore, to predict the spread area of repeated ASF outbreaks on a long-term basis, a spatiotemporal analysis of the ASF outbreak needs to be performed in conjunction with climate change scenarios in further studies.

### 4.2. Shortest-Path for ASF Outbreaks in Wild Boars

Animal movement networks play a critical role in understanding the spread of infectious diseases through wildlife vectors such as ASF [94]. Using shortest-path BC analysis to predict the propagation path of ASF in wild boars, we found that 66% of the 252 ASF outbreaks in the field-evaluation dataset were within a range of 1.8 km from the shortest path, and 92% were within a range of 3.6 km. The frequency of ASF outbreaks per km^2^ was 2.5 times higher in the range of 1.8 km than in the range of 1.8–3.6 km. Thus, we can confirm that the shortest-path map of the ASF outbreak generated in this study is useful in predicting the locations of ASF outbreaks in wild boars and a high-risk area for ASF outbreaks is predicted within 1.8 km of the path.

Most of the shortest paths of the ASF outbreak were distributed along mountain ranges (Baekdudaegan and Jeongmaeks) and were consistent with the locations of wild boar ASF outbreaks in 2014–2018 [64,95]. Among the main paths that run from north to south in South Korea, wild boar ASF outbreaks were high along the path connecting R1 to I8 via R5 in the central are, and the path with R7 and I11 was along the NDJM in the east. Among the main paths across South Korea from east to west, wild boar ASF outbreaks were high in the paths from I4 in the west to I11 via I8 and to Gyeongju National Park, located below I11 in the east.

ASF has spread to R5 from GW but has not yet spread to I4, I5, and I6 in the west of R5; I7, I8, I9, and I10 in the middle; or R7 and I11 in the east. ASF has not yet spread to three locations, R7, I8, and I9, located south of R5. Of the seven national parks near VHKD locations, ASF has not yet spread to four (Deogyusan National Park, Gayasan National Park, Gyeryongsan National Park, and Juwangsan National Park) or those in close proximity to the VHKD locations (I8, I10, I6, and R7). If VHKD locations are invaded, they are likely to serve as hubs for the spread of ASF; therefore, they should be included in the priority areas for controlling wild boar populations and searching for carcasses in advance.

### 4.3. Pig Farming Sectors at High Risk of ASF Spillover and Its Management Implications

After the first outbreak of ASF on September 16, 2019, in South Korea [23], the disease has been limited to only 0.59% of 5063 pig farms, with a few outbreaks in some localities in GG and GW [67]. However, the disease has begun to spread widely among wild boars to the south of GW, which increases the risk of ASF spillover to the pig farm sector near mountain ranges.

Predicting suitable areas for ASF outbreaks in wild boars is essential in determining management strategies and policies for preemptive quarantine of ASF. This is achieved by specifying search areas for the rapid detection and removal of ASF-infected carcasses, fence installation, and hunting [23,96,97], particularly in mountainous countries such as South Korea. To date, studies on the spread of ASF in South Korea have been limited to ASF-affected areas [1,98], some areas adjacent to ASF-affected areas [98], and the potential growth trend of ASF-affected areas [64]. However, no studies have predicted the future distribution of ASF outbreaks in wild boars on a nationwide scale.

This study predicted areas with a high probability of ASF outbreaks in wild boars and specified pig farm sectors at high risk of ASFV spillover, located in areas with a high possibility of ASF outbreaks in wild boars. Using the field-evaluation dataset, we compared the model results with the location of ASF outbreaks in wild boars in local regions (CB and GG) where ASF is newly spreading and, based on the results of the field evalution, predicted area with a high risk of ASF outbreak on a national level (areas within 1.8 km from the shortest path and areas with a suitability value ≥ 0.4). Among the 5063 pig farms, 9 individual farms and 14 pig farm sectors were located in high-risk zones in both the shortest-path and suitability maps.

In South Korea, mountain forests account for 63% of the land area [99], which includes the Baekdudaegan Mountain Range and nine mountain ranges that branch off from the Baekdudaegan Mountain Range [100]. Wild boars primarily inhabit mountain forests and can damage crops during cultivation [101,102]. Mountain ranges render the systematic search for wild boar carcasses difficult, resulting in them becoming a significant channel for the spread of ASF in wild boars in South Korea [23]. ASFV-infected carcasses serve as ASFV reservoirs and mediums for ASF transmission between wild boars and between wild boars and domestic pigs [103,104,105,106,107]. Therefore, regular and strengthened carcass searches, particularly around the 11 pig farm sectors in the high-risk zones adjacent to mountain ranges, are required as essential quarantine measures to prevent ASF spillover into pig farm sectors.

In South Korea, search teams have been organized and operated by the Korea National Park Service and local environment agencies to search for wild boar carcasses in forests [67]. However, appropriate standards for selecting the geographical range of the search have not been established. Searching for carcasses in a wide range of mountain forests in mountainous countries such as South Korea requires considerable time and cost. Therefore, it is necessary to limit the range of the search to areas with a high possibility of carcass detection to increase search efficiency. This study identified a high-risk area for ASF outbreaks, contributing towards establishing priorities for searching areas for carcasses and controlling wild boar populations. Additionally, identifying pig farms with a high risk of ASFV spillover through wild boar carcasses helps to preemptively establish quarantine measures, such as strengthening biosecurity within pig farms and searching for carcasses in the surrounding forests.

## 5. Conclusions

Our study has facilitated the selection of a priority range for searching for wild boar carcasses by accurately predicting the specific pathways of ASF outbreaks in wild boars. Furthermore, by linking the prediction model of ASF outbreaks in wild boars with the distribution of pig farms, we have successfully identified pig farms that face a high risk of ASF spillover from wild boars. This significant contribution has enabled the establishment of preemptive farm-centered quarantine measures.

This study highlights the need for field evaluation as well as model self-tests for the evaluation of model quality, such as comparing the frequency of actual ASF outbreaks in areas predicted to be potentially high and low risk by models, when evaluating a prediction model for a rapidly spreading disease such as ASF. The field evaluation contributes significantly to verifying and more specifying the model’s results. The results of MaxEnt model and shortest-path BC analysis combined with the field evaluation predicted a high likelihood of ASF outbreaks in areas with a suitability value of 0.4 or higher and within a range of 1.8 km from the shortest path. These ranges could be defined as high-risk zones for ASF outbreak, particularly in the early stages of its spread. Of the 5063 pig farms in South Korea, 9 pig farms and 14 pig farm sectors with a kernel density ≥ 8 were located in both high-risk zones identified by the MaxEnt model and the shortest-path BC analysis.

This study provides critical insights for preventing and controlling ASF outbreaks. Predicting suitable areas and paths for ASF outbreaks can help to implement preemptive measures to minimize the impact of ASF on wild boars and pig farms. Improved biosecurity measures and regular carcass searches around pig farm sectors at high risk can help mitigate the risk of ASF spillover from wild boars.

The spatial range of our study was limited to South Korea and was designed to predict the extent of ASF spread within a relatively short timeframe. Therefore, we did not include climate factors as variables in our analysis. Given the global spread of ASF and repeated ASF outbreaks on a long-term basis, future studies should include weather variables, including climate change scenarios, to establish comprehensive long-range prediction models that extend beyond the borders of Korea and cover larger geographic areas. This study, based on the South Korean situation, which directly links the potential spread routes of ASF predicted by theoretical models with the given distribution of pig farms, shows how scientific research can contribute to establishing ASF quarantine policies and will be a reference for epidemiologists, ecologists, and policy makers in countries where ASF occurs.

## Figures and Tables

**Table 1:** Variables selected for modeling.

Category	Variable	Abbreviation	Source
Forest environment	Forest type	Frtp	Forest Type Map (1:5000) from Korea Forest Service (accessed on 24 November 2021) https://www.forest.go.kr/
	Age classes of trees	Agcl	
	Diameter at breast height of trees	Dbht	
	Crown density	Crde	
Topographic	Elevation	Elev	Digital Elevation Model from National Geographic Information Institute of the Republic of Korea (accessed on 5 November 2021) http://data.nsdi.go.kr/
	Slope	Slop	
	Aspect	Aspe	
	Water	Water	Stream Order Map from Water Resources Management Information System of the Republic of Korea (accessed on 23 November 2021) http://www.wamis.go.kr/
Anthropogenic	Settlement	Setl	Subdivision Land Cover Map from Ministry of Environment of the Republic of Korea (accessed on 25 November 2021) https://egis.me.go.kr/
	Plow	Plow	
	Road	Road	Road map of Korea from Ministry of Land of the Republic of Korea (accessed on 21 November 2021) https://www.its.go.kr/

**Table 2:** Suitable areas for ASF outbreaks in CB and GB predicted using the MaxEnt model and the distribution of 252 ASF outbreaks in the field-evaluation dataset.

Level of Suitability Value	Area (km^2^) at Suitability Levels	Percentage of Area in Each Suitability Level out of the Total Area (16,469.1 km^2^)	No. of ASF OutBreaks at Suitability Level	Percentage of ASF Outbreaks at Suitability Level out of the Total ASF Outbreaks (252 Cases)	No. ASF OutBreaks/km^2^
Very high (0.8–1)	15.8	0.1	2	0.8	0.127
High (0.6–0.8)	760.7	4.6	59	23.4	0.078
Medium (0.4–0.6)	4047.7	24.6	112	44.4	0.028
Low (0.2–0.4)	7482.4	45.4	73	29.0	0.010
Very low (0–0.2)	4162.5	25.3	6	2.4	0.001
Total	16,469.1	100	252	100	0.015

**Table 3:** Distribution of the 252 ASF outbreaks in the field-evaluation dataset based on the distance from the shortest-path.

Distance from Shortest-Path	Level of Shortest-Path BC	Area Size (km^2^)	No. of ASF Outbreaks	No. of ASF Outbreaks/km^2^
1.8 km	Low (0.007~0.026)	3329	87	0.026
	Medium (0.026~0.064)	1457	43	0.030
	High (0.064~0.149)	505	37	0.073
1.8~3.6 km	Low (0.007~0.026)	3373	26	0.008
	Medium (0.026~0.064)	1312	34	0.026
	High (0.064~0.149)	394	6	0.015

**Table 4:** Information on 51 pig farm sectors with farm density of average kernel density of 0.8 or higher.

Local Region	Pig Farm Sector	Locality	No. of Pig Farms	No. of Heads	Distance from the Shortest Path (km)	Overlap Area * (km^2^)
Gyeonggi-do (GG)	GG-1	Yeoncheon-gun	10	17,555	5.8	
	GG-2	Pocheon-si	28	82,880	5.9	0.11
	GG-3	Pocheon-si	14	14,735	8.6	0.56
	GG-4	Dongducheon-si **	13	24,140	1.2	0.01
	GG-5	Yangju-si	13	25,720	5.0	
	GG-6 *	Yangpyeong-gun	10	21,710	0.0	0.49
	GG-7	Icheon-si	10	17,340	6.0	0.00
	GG-8 *	Cheoin-gu **	13	9932	0.0	0.38
	GG-9	Icheon-si **	12	33,795	15.5	
	GG-10	Yeoju-si	4	6024	11.5	
	GG-11	Icheon-si	8	14,900	10.9	0.00
	GG-12	Cheoin-gu **	216	364,060	0.6	1.93
	GG-13	Pyeongtaek-si **	10	6373	11.9	
	GG-14 *	Anseong-si	12	17,700	0.0	0.02
	GG-15	Pyeongtaek-si **	21	48,546	14.8	
Gangwon-do (GW)	GW-1	Cheorwon-gun **	10	36,414	25.5	
Chungcheongbuk-do (CB)	CB-1	Goesan-gun	9	14,490	0.0	
	CB-2	Cheongwon-gun **	5	10,195	3.9	
Chungcheongnam-do (CN)	CN-1	Dangjin-si	12	16,785	12.5	
	CN-2	Dongnam-gu **	8	25,830	3.8	0.08
	CN-3	Asan-si	2	3200	16.6	
	CN-4	Dangjin-si **	36	72,427	6.7	
	CN-5	Asan-si **	2	6000	12.7	
	CN-6	Yesan-gun	17	36,004	11.6	
	CN-7 *	Hongseong-gun **	16	39,220	0.0	0.02
	CN-8	Hongseong-gun	1	2000	0.0	
	CN-9 *	Boryeong-si **	285	604,359	0.0	0.80
	CN-10	Gongju-si	4	6550	0.0	
	CN-11	Gongju-si **	34	33,483	2.9	
	CN-12	Nonsan-si	25	37,540	9.9	1.12
Sejong-si (SJ)	SJ-1	Yuseong-gu **	13	26,230	13.6	0.35
Jeollabuk-do (JB)	JB-1	Iksan-si	21	19,750	3.1	
	JB-2	Gunsan-si **	13	22,900	0.0	
	JB-3	Iksan-si **	97	80,999	8.9	
	JB-4	Gimje-si	11	25,630	13.4	
	JB-5	Gimje-si **	66	133,895	16.1	
Jeollanam-do (JN)	JN-1	Hwasun-gun	9	13,773	8.3	0.32
	JN-2	Naju-si	5	16,150	12.2	
	JN-3	Muan-gun	9	16,150	3.0	
Gwangju-si (GJ)	GJ-1	Gwangsan-gu **	30	38,482	11.9	
Gyeongsangbuk-do (GB)	GB-1	Yeongcheon-si	12	28,900	4.7	0.03
	GB-2 *	Yeongcheon-si	4	7400	0.0	0.21
	GB-3 *	Seongju-gun	12	12,860	0.0	1.34
	GB-4 *	Gyeongju-si **	21	29,985	0.0	0.01
	GB-5	Gyeongsan-si	11	20,442	8.5	
	GB-6 *	Hapcheon-gun **	10	26,200	0.0	0.04
Gyeongsangnam-do (GN)	GN-1	Hapcheon-gun **	20	33,510	0.1	1.80
	GN-2	Miryang-si	13	10,280	6.0	0.35
	GN-3	Yangsan-si	13	17,896	4.0	0.44
	GN-4	Gimhae-si	57	97,584	0.1	1.26
	GN-5 *	Goseong-gun	12	18,578	0.0	0.10

* Pig farm sectors overlapping or adjacent (0 km) to areas with a suitability value of 0.4 or higher. ** indicates sectors are distributed across two or more localities.

## Data Availability

The data are available upon request from the corresponding author.

## Supplementary Materials

The following supporting information can be downloaded at: https://www.mdpi.com/article/10.3390/ani13132148/s1, Figure S1: Geographical distribution of the Korean mountain ranges comprising the Baekdudaegan Mountain Range (Baekdugaegan), an elongated ridge that runs from Mt. Baekdu-san in North Korea to Jirisan National Park in South Korea, and the nine mountain ranges (Jeongmaeks) that branch off from the Baekdudaegan [100]. Civilian Control Line (CCL), which, located 10 km south along the Military Demarcation Line (MDL), is intended to control citizen access. The area between MDL and CCL is called the Civilian Control Area (CCA); Figure S2: Map for each environmental variable; Figure S3: Area Under the ROC Curve (AUC) value, which is calculated from the area under the Receiver Operating Characteristic Curve obtained through 10 bootstrap replicates; Figure S4: Geographical distribution of 37 individual pig farms located in an area with a suitability value of 0.4 or higher; Figure S5: Geographical distribution of 499 pig farms within a 1.8-km range of the shortest-path; Table S1: Standardized protocol of the SDM process, following the ODMAP v1 [70]; Table S2: Relative importance of variables used in the MaxEnt model.
