# Supplementary material for: Predicting Suitable Areas for African Swine Fever Outbreaks in Wild Boars in South Korea and Their Implications for Managing High-Risk Pig Farms
Source: Animals (Basel). 2023 Jun 29;13(13):2148. doi: 10.3390/ani13132148 (PMC10339976; doi:10.3390/ani13132148)
Supplement: Supplementary file 1 [file animals-13-02148-s001.zip › animals-2412387-supplementary.pdf]

Supplementary Materials

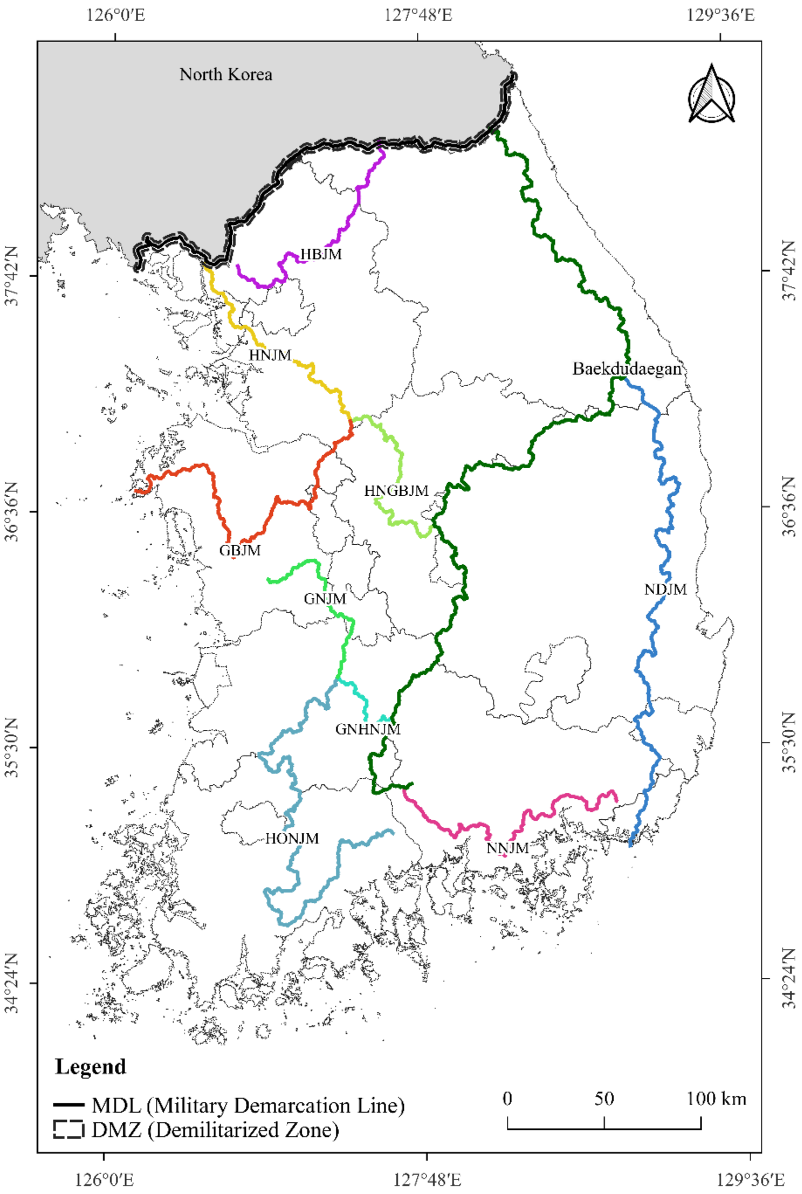

Figure S1. Geographical distribution of the Korean mountain ranges comprising the Baekdudaegan Mountain Range (Baekdugaegan), an elongated ridge that runs from Mt. Baekdu-san in North Korea to Jirisan National Park in South Korea, and the nine mountain ranges (Jeongmaeks) that branch off from the Baekdudaegan [103]. Civilian Control Line (CCL), which, located 10 km south along the Military Demarcation Line (MDL), is intended to control citizen access. The area between MDL and CCL is called the Civilian Control Area (CCA).

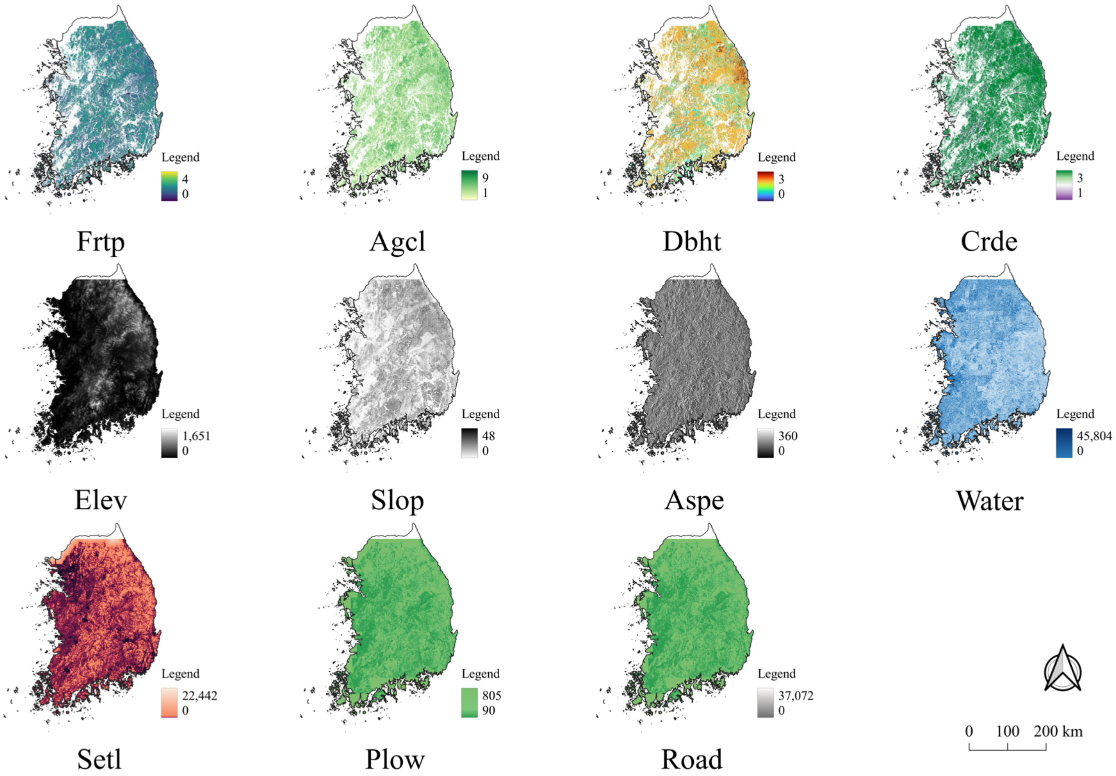

Figure S2. Map for each environmental variable.

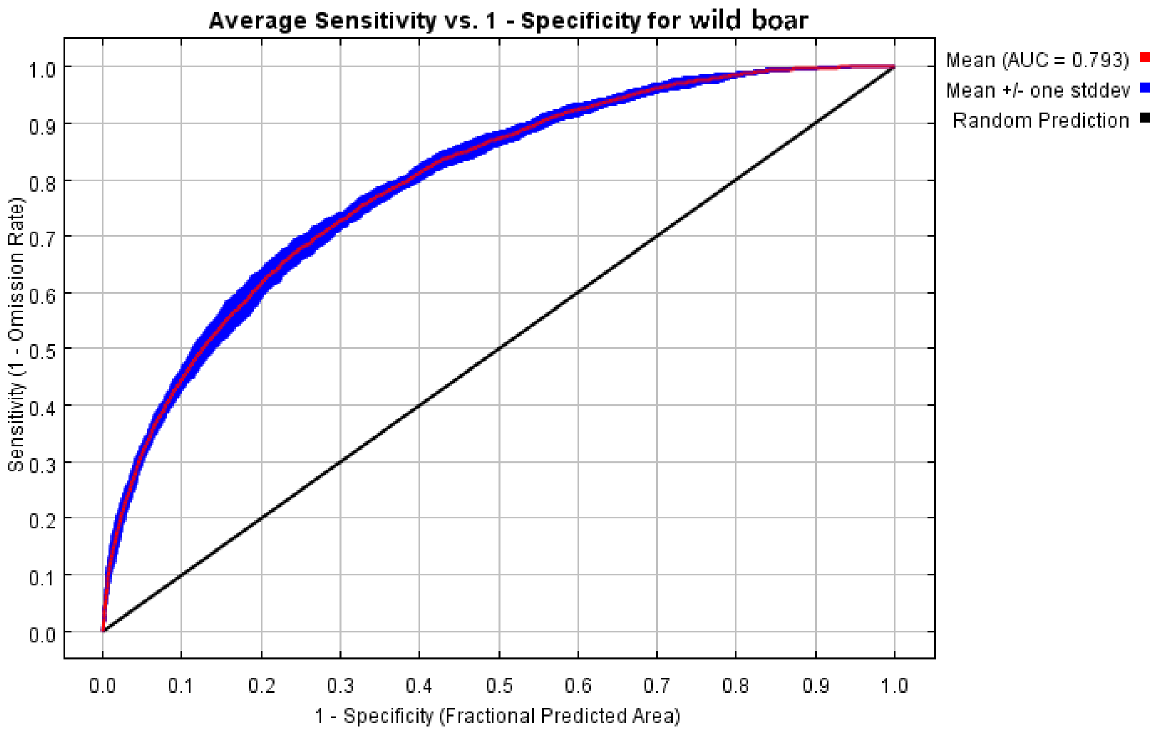

Figure S3. Area Under the ROC Curve (AUC) value, which is calculated from the area under the Receiver Operating Characteristic Curve obtained through 10 bootstrap replicates.

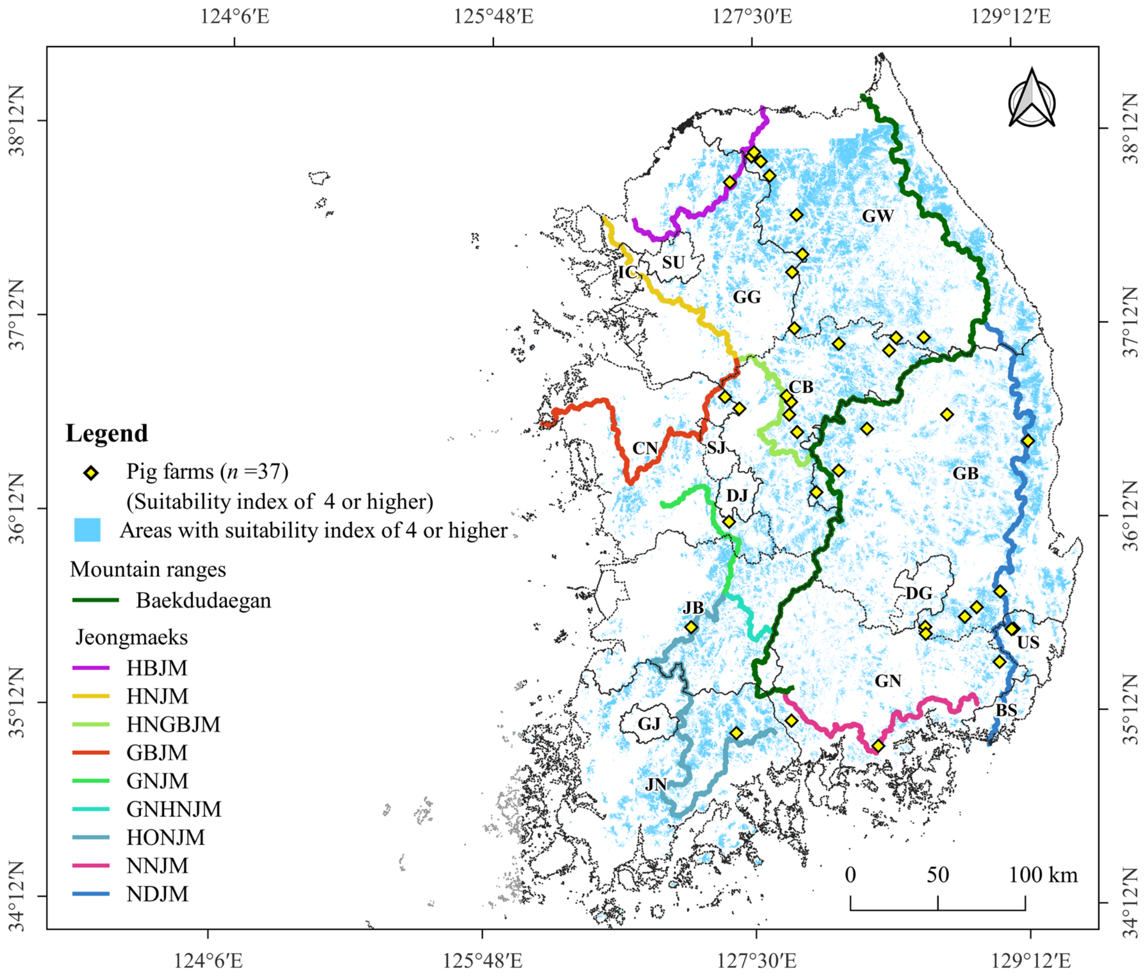

Figure S4. Geographical distribution of 37 individual pig farms located in an area with a suitability value of 0.4 or higher.

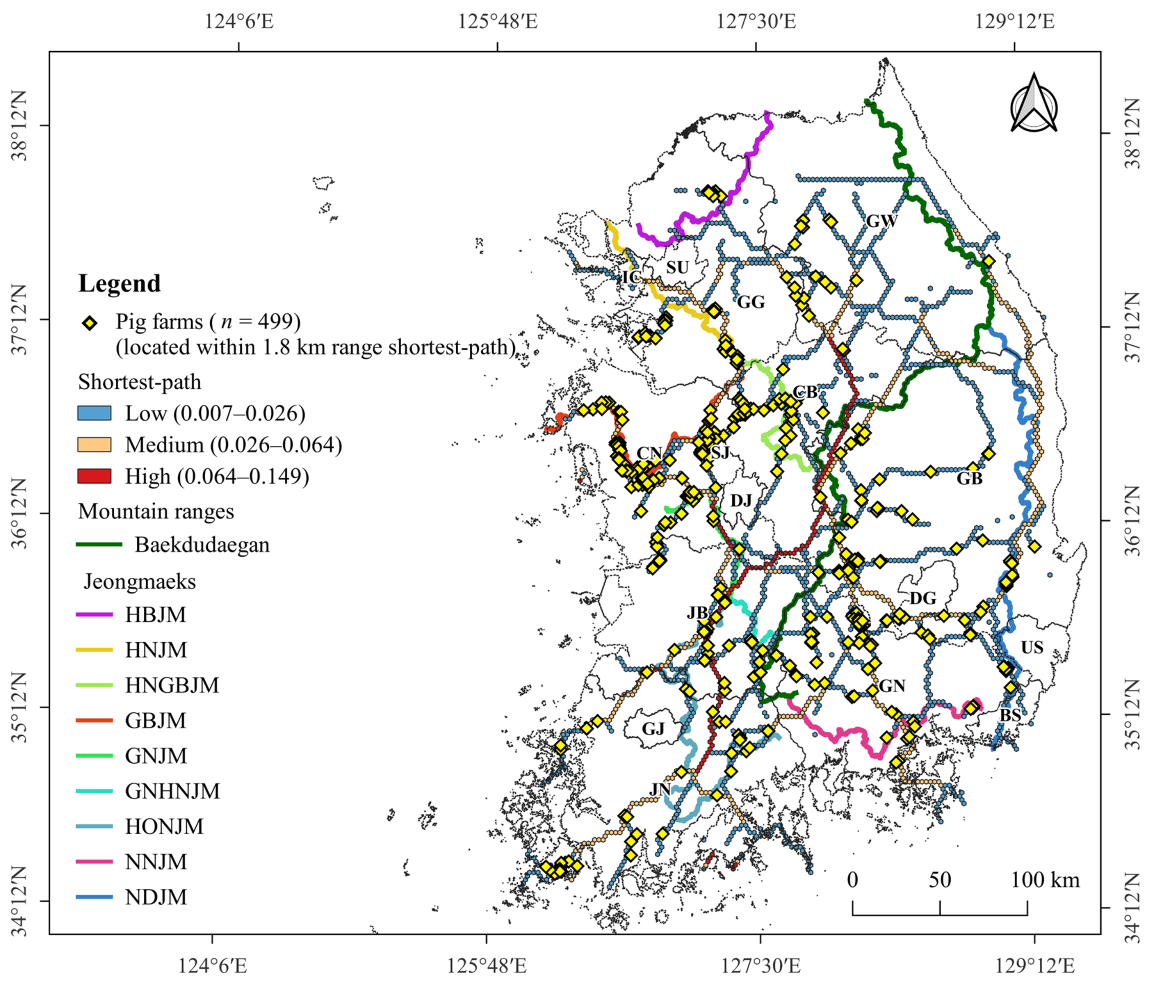

Figure S5. Geographical distribution of 499 pig farms within a 1.8-km range of the shortest-path.

Table S1. Standardized protocol of the SDM process, following the ODMAP v1 [72].

Overview

Model objective
Model objective: Forecast and transfer
Target output: Probability of ASF outbreaks in wild boar
Focal Taxon
Focal Taxon: *Sus scrofa*
Location
Location: South Korea
Scale of Analysis
Spatial extent: 125.78, 129.66, 34.13, 38.25 (xmin, xmax, ymin, ymax)
Spatial resolution: 90m
Temporal extent: 2019~2021
Temporal resolution: 90m
Boundary: natural, political
Biodiversity data
Observation type: field survey
The data was collected through intensive surveys by wild boar carcass search teams and wild boar hunting teams over a period of approximately two years, from September 2019 to November 2021.
Response data type: presence-only
Predictors
Predictor types: habitat, topographic
Hypotheses
Hypotheses: ASF-infected wild boar are affected by habitat type and topography
Assumptions
Model assumptions: There is no sampling bias in the ASF occurrence data, as the data were collected by intensively surveying ridges, hills, and valleys in a wide area for two years.
Algorithms
Modelling techniques: maxent
Model complexity: The data was randomly divided into 80% training data set and 20% test data set.
Model averaging: The final prediction model for the ASF outbreak was selected as the mean model from ten bootstrap replicates.
Workflow
Model workflow: We randomly divided the 591 collected data into training data (80%, n=473) and testing data (20%, n=118). We set the background points to 20,000 and selected the average model from the models obtained by 10 bootstrap iterations. The model was validated using AUC and TSS.
Software
Software: MaxEnt 3.4.4 software
Code availability: no have
Data availability: no have

Data

Biodiversity data
Taxon names: *Sus scrofa*
Taxonomic reference system: GBIF Backbone Taxonomy
Ecological level: species
Data sources: Coordinates of ASF outbreaks: https://www.mafra.go.kr/FMD-AI2/2145/subview.do
Sampling design: random

Sample size: prevalence

Data partitioning

Training data: training dataset 80%

Validation data: testing dataset 20%

Predictor variables

Predictor variables: Forest type, Age classes of trees, Diameter at breast height of trees, Crown density, Elevation, Slope, Aspect, Water, Settlement

Data sources: Forest Type Map (1:5000) from Korea Forest Service (accessed on 24 November 2021, https://www.forest.go.kr/) Digital Elevation Model from National Geographic Information Institute of the Republic of Korea (accessed on 5 November 2021, http://data.nsdi.go.kr/) Stream Order Map from Water Resources Management Information System of the Republic of Korea (accessed on 23 November 2021, http://www.wamis.go.kr/) Subdivision Land Cover Map from Ministry of Environment of the Republic of Korea (accessed on 25 November 2021, https://egis.me.go.kr/) Road map of Korea from Ministry of Land of the Republic of Korea (accessed on 21 November 2021, https://www.its.go.kr/)

Spatial extent: 125.78, 129.66, 34.13, 38.25 (xmin, xmax, ymin, ymax)

Spatial resolution: 90m

Coordinate reference system: EPSG:5181 - Korea 2000 / Central Belt

Temporal extent: 2019~2021

Model

Multicollinearity

Multicollinearity: Correlation and collinearity were analyzed to produce a reliable and unbiased model of species distribution. We evaluated the correlations between the 11 environmental variables using Spearman’s correlation coefficient in R version 4.2.2. None of the variables showed a correlation (absolute value less than 0.75) or multicollinearity.

Model settings

Model settings (extrapolation): We uses presence-only data and 20,000 randomly generated background points. All other parameters were set to their default values (Maximum iterations = 500, Convergence threshold = 0.00001, Default prevalence = 0.5) (Elith et al., 2011)

Model estimates

Coefficients: The relative importance of variables used in the MaxEnt model is shown in Table S1.

Analysis and Correction of non-independence

Spatial autocorrelation: Data sets for testing and training were not spatially autocorrelated (Moran's I test, p-value: 0.973) and therefore all occurrence records were retained for spatial analyses.

Threshold selection

Threshold selection: The continuous prediction was further classified into five classes (0–0.2: very low suitability, 0.2–0.4: low suitability, 0.4–0.6: medium suit-ability, 0.6–0.8: high suitability, 0.8–1: very high suitability) (Yang et al., 2013; Ma et al., 2018)

Assessment

Performance statistics

Performance on training data: AUC, TSS

Performance on test data: AUC, TSS

Performance on validation data: We validated using a field-evaluation dataset.

Plausibility check

Response shapes: partial response plots

Expert judgement: map display

Prediction

Prediction output

Prediction unit: ASF outbreak suitability value

Table S2. Relative importance of variables used in the MaxEnt model.

Variable	Percent contribution	Permutation importance
Elevation	33	25.7
Road	16	12.8
Settlement	15.6	18.2
Aspect	9.4	9.2
Plow	6.8	12.6
Water	6.1	6
Slope	5.4	7.6
Age classes of trees	3.1	3.6
Forest type	2.8	2.6
Crown density	0.9	0.4
Diameter at breast height of trees	0.8	1.2
